# Letter in Response of the Article “Postural Hypervigilance and Perception of Correct Sitting Posture in Individuals with and without Low Back Pain”

**DOI:** 10.1055/s-0044-1779322

**Published:** 2024-03-21

**Authors:** Eduardo Lima de Oliveira, Paula Fernanda Ferreira Coutinho, Uiara Martins Braga, Leonardo Drumond Barsante

**Affiliations:** 1 Faculdade Ciências Médicas de Minas Gerais (FCM-MG), Belo Horizonte, Minas Gerais, Brasil

I would like to express my gratitude to the editor for the letter sent to us, which is filled with compliments and information. The content is so relevant that it allows us to gain a broader perspective on the topic of postural hypervigilance and the perception of correct siting posture in individuals with and without low back pain, as well as its association with other types of back pain. This approach is crucial because, aside from being different, it presents specific characteristics and considerations.

For instance, neck pain is common and can be associated with a variety of factors, including poor posture, muscle tension, and injuries. Similar to low back pain, the perception of correct posture plays an important role in the prevention and treatment of neck pain. In these cases, postural hypervigilance may also be present, leading to increased stress in the cervical region and worsening of symptoms. 1 2

Likewise, back pain may be related to postural problems and muscle tension in the middle back region. Correct perception of sitting posture and awareness about maintaining good posture can be useful in preventing and treating this type of back pain. 3 4

Therefore, it is important to consider the relationship between postural hypervigilance, perception of correct posture and different types of back pain, including neck pain and dorsal pain. Integrated approaches involving postural education, specific exercises and behavior modification strategies can benefit individuals with different types of back pain, helping them improve their posture, alleviate pain and promote a better quality of life.

I appreciate the opportunity to share this perspective and hope that this additional information contributes to the discussion about postural hypervigilance and perception of correct posture in relation to different types of back pain and the emergence of future studies.
