# Supplementary material for: Letter in Response of the Article “Postural Hypervigilance and Perception of Correct Sitting Posture in Individuals with and without Low Back Pain”
Source: Rev Bras Ortop (Sao Paulo). 2024 Mar 21;59(1):e150–2. doi: 10.1055/s-0044-1779322 (PMC10957265; doi:10.1055/s-0044-1779322)
Supplement: Related texts

# Research article: Carta resposta referente ao artigo “Hipervigilância postural e percepção da postura correta sentada em indivíduos com e sem dor lombar”

Gostaria de agradecer ao editor a carta que nos foi enviada com todos os elogios e informações tão pertinentes que nos possibilita ter uma maior perspectiva em relação ao tema da hipervigilância postural e percepção da postura correta em indivíduos com e sem dor lombar, assim como sua associação com outros tipos de dor nas costas. Essa abordagem é crucial, pois, além de serem diferentes, apresentam características e considerações específicas.

A dor cervical, por exemplo, é comum e pode estar associada a uma variedade de fatores, incluindo postura inadequada, tensão muscular e lesões. Assim como na dor lombar, a percepção da postura correta desempenha um papel relevante na prevenção e no tratamento da dor cervical. A hipervigilância postural nesses casos também pode estar presente, levando a um maior estresse na região cervical e agravando os sintomas. 1 2

Da mesma forma, a dor dorsal pode estar relacionada a problemas posturais e tensão muscular na região média das costas. A percepção correta da postura sentada e a conscientização sobre a manutenção de uma boa postura podem ser úteis na prevenção e no tratamento desse tipo de dor nas costas. 3 4

Portanto, é importante considerar a relação entre hipervigilância postural, percepção da postura correta e diferentes tipos de dor nas costas, incluindo dor cervical e dor dorsal. Abordagens integradas que envolvam educação postural, exercícios específicos e estratégias de modificação de comportamento podem beneficiar indivíduos com diferentes tipos de dor nas costas, ajudando-os a melhorar sua postura, aliviar a dor e promover uma melhor qualidade de vida.

Agradeço a oportunidade de compartilhar essa perspectiva e espero que essas informações adicionais contribuam para a discussão sobre hipervigilância postural e percepção da postura correta em relação a diferentes tipos de dor nas costas e no surgimento de futuros estudos.
